# Patterns of Head CT Utilization in Emergency Department Patients With Minor Head Injury: A Systematic Review

**DOI:** 10.7759/cureus.94370

**Published:** 2025-10-11

**Authors:** Satyasuna Kafle, Jaipal Dass, Roshan Shrestha, Dipesh Karki, Saif Abdulsattar, Hassan Imtiaz, Areeba Zahid, Muhammad Rizwan Umer, Havil Stephen Alexander Bakka, Inam Rafiq

**Affiliations:** 1 Acute Medicine, Queen's Hospital, Barking, Havering and Redbridge University Hospitals NHS Trust (BHRUT), Romford, GBR; 2 Emergency Medicine, Walsall Healthcare NHS Trust, Walsall, GBR; 3 Stroke Medicine, Barking, Havering and Redbridge University Hospitals NHS Trust, London, GBR; 4 Vascular Surgery, Northwick Park Hospital, London, GBR; 5 Trauma and Orthopedics, Manchester University NHS Foundation Trust, Manchester, GBR; 6 Trauma and Orthopedics, University Hospitals Dorset NHS Foundation Trust, Poole, GBR; 7 Medicine, Faisalabad Medical University, Faisalabad, PAK; 8 Trauma and Orthopedics, Royal Sussex County Hospital, Brighton and Hove, GBR; 9 Neurosurgery, Royal Sussex County Hospital, Brighton and Hove, GBR; 10 Internal Medicine, Dow University of Health Sciences, Civil Hospital Karachi, Karachi, PAK

**Keywords:** canadian ct head rule, emergency department, head ct, imaging utilization, minor head injury, new orleans criteria, nexus-ii, nice guidelines

## Abstract

Minor head injury (MHI) is a frequent presentation to emergency departments (EDs), and while most patients recover uneventfully, a small proportion develop clinically important traumatic brain injury (ciTBI). Computed tomography (CT) is the diagnostic gold standard for detecting intracranial pathology, but its widespread use contributes to unnecessary radiation exposure, higher costs, and ED crowding. To optimize utilization, several clinical decision rules, including the Canadian CT Head Rule, New Orleans Criteria, National Emergency X-Radiography Utilization Study II (NEXUS-II), and National Institute for Health and Care Excellence (NICE) guidelines, have been developed to balance sensitivity for ciTBI with the need to limit avoidable scans. This systematic review, conducted according to Preferred Reporting Items for Systematic Reviews and Meta-Analyses (PRISMA) methodology, searched PubMed, Embase, Scopus, and the Cochrane Library up to August 2025. Of the 96 records screened, five studies with over 44,000 patients met the inclusion criteria. The findings indicate that although decision rules demonstrate high sensitivity and strong potential to reduce unnecessary imaging, variability in adherence leads to both overuse and underuse of CT. Greater integration of validated rules into clinical workflows and decision-support systems is needed to enhance patient safety, reduce costs, and improve efficiency in the management of MHI.

## Introduction and background

Minor head injury (MHI), often referred to as mild traumatic brain injury (m-TBI), is one of the most frequent reasons for emergency department (ED) visits worldwide. It is estimated that more than 50 million individuals sustain a traumatic brain injury annually, with 70-90% classified as mild in severity [1]. Although most patients present with Glasgow Coma Scale (GCS) scores of 13-15 and recover uneventfully, a subset may harbor clinically important intracranial lesions requiring acute intervention. The challenge for clinicians lies in identifying these high-risk patients while avoiding unnecessary imaging in those with trivial injuries. Computed tomography (CT) of the head remains the gold standard for rapid evaluation of patients with suspected intracranial injury. Its widespread availability and diagnostic accuracy have made it indispensable in acute trauma care [2].

However, CT use carries the following important drawbacks: exposure to ionizing radiation, incidental findings leading to further testing, ED crowding, and significant healthcare costs [3]. Concerns are particularly heightened in younger patients, where cumulative radiation exposure increases the lifetime risk of malignancy. These considerations highlight the need for judicious, evidence-based use of CT in MHI patients. To optimize resource utilization, multiple evidence-based guidelines and decision rules have been proposed. The World Health Organization (WHO) and the Centers for Disease Control and Prevention (CDC) emphasize structured clinical assessment as the cornerstone of m-TBI management [4]. Moreover, systematic reviews show that when clinical decision instruments are consistently applied, unnecessary CTs can be reduced without compromising patient safety [5]. Despite this, studies demonstrate persistent variability in CT ordering practices among physicians, influenced by medico-legal concerns, patient expectations, and institutional protocols.

Global practice patterns reflect substantial differences in CT use. High-income countries report rising imaging rates despite validated guidelines, while resource-limited settings face challenges in access, leading to underutilization and delayed diagnosis. The gap between evidence and practice underlines the importance of understanding real-world imaging trends, barriers to guideline adherence, and their impact on patient outcomes. By reviewing the patterns of CT utilization in MHI, we can better inform policy, refine decision support, and ultimately improve the safety and efficiency of emergency care. The aim of this systematic review is to evaluate patterns of head CT utilization in emergency department patients presenting with minor head injury, with a focus on adherence to clinical decision rules, diagnostic yield, and implications for patient safety and healthcare resources.

## Review

Materials and methods

Search Strategy

A systematic literature search was conducted in PubMed, Embase, Scopus, and the Cochrane Library from inception through August 2025. Search terms included combinations of “minor head injury,” “mild traumatic brain injury,” “head CT,” “computed tomography,” “Canadian CT Head Rule,” “New Orleans Criteria,” “NEXUS-II,” and “NICE guidelines.” Reference lists of included articles were also screened to identify additional relevant studies. The search adhered to Preferred Reporting Items for Systematic Reviews and Meta-Analyses (PRISMA) guidelines [6].

Eligibility Criteria

This review applied the PICO framework to define eligibility criteria [7]. The population (P) included adult patients aged 16 years or older presenting to the emergency department with minor head injury, typically defined as a Glasgow Coma Scale score of 13-15. The intervention or exposure (I) was the use of head CT imaging, either performed directly or evaluated for appropriateness. The comparator (C) was physician clinical judgment or the application of structured clinical decision rules, including the Canadian CT Head Rule (CCHR), New Orleans Criteria (NOC), or New Orleans Criteria, National Emergency X-Radiography Utilization Study II (NEXUS-II) criteria. The outcomes (O) of interest included CT utilization rates, diagnostic yield, detection of clinically important traumatic brain injury (ciTBI), need for neurosurgical intervention, and adherence to guideline recommendations. Eligible studies were prospective or retrospective cohort studies, multicenter trials, or audit-based investigations conducted in emergency care settings. Exclusion criteria were case reports, animal studies, editorials, and conference abstracts, as these did not provide systematic evidence relevant to the review objective.

Study Selection

The study selection process followed the PRISMA guidelines. Two independent reviewers systematically screened the titles and abstracts of all records identified through database searching. Articles that appeared potentially relevant were retrieved in full and assessed using the predefined inclusion and exclusion criteria. Any disagreements during the screening or eligibility assessment were resolved through consensus, and if consensus could not be reached, a third senior reviewer adjudicated the decision.

Data Extraction

A standardized data extraction form was developed and used by both reviewers to collect relevant information from the included studies. Extracted data included study design, setting, population characteristics, and the specific clinical criteria or decision rules evaluated. In addition, details regarding CT utilization rates, diagnostic yield, frequency of clinically important traumatic brain injury (ciTBI), and adherence to guideline recommendations were recorded. The extraction process also captured study limitations and notable findings that could influence interpretation. All data were cross-verified to maintain consistency and accuracy.

Risk of Bias Assessment

The risk of bias in the included studies was assessed using established frameworks. For prospective and retrospective cohort studies, the Newcastle-Ottawa Scale (NOS) was applied, which evaluates domains such as selection of participants, comparability of cohorts, and ascertainment of outcomes [8]. For audit-based studies, adapted observational criteria were used to evaluate methodological rigor. Each study was rated as having a low, moderate, or high risk of bias based on transparency of reporting, methodological strength, and susceptibility to confounding. The use of structured appraisal tools allowed for a transparent evaluation of study quality and provided context for interpreting the review findings.

Data Synthesis

Given the heterogeneity of study designs, populations, and outcome measures, a quantitative meta-analysis was not feasible. Instead, a narrative synthesis approach was undertaken. The extracted data were organized into comparative tables to highlight key features, including population demographics, decision rules applied, CT utilization rates, diagnostic yield, and clinical outcomes. Results were then thematically synthesized to identify patterns in CT use, evaluate adherence to clinical decision rules, and highlight common sources of variation across healthcare systems. The synthesis emphasized both the clinical and policy implications of CT use in minor head injury.

Results

Study Selection Process

**Figure 1:**
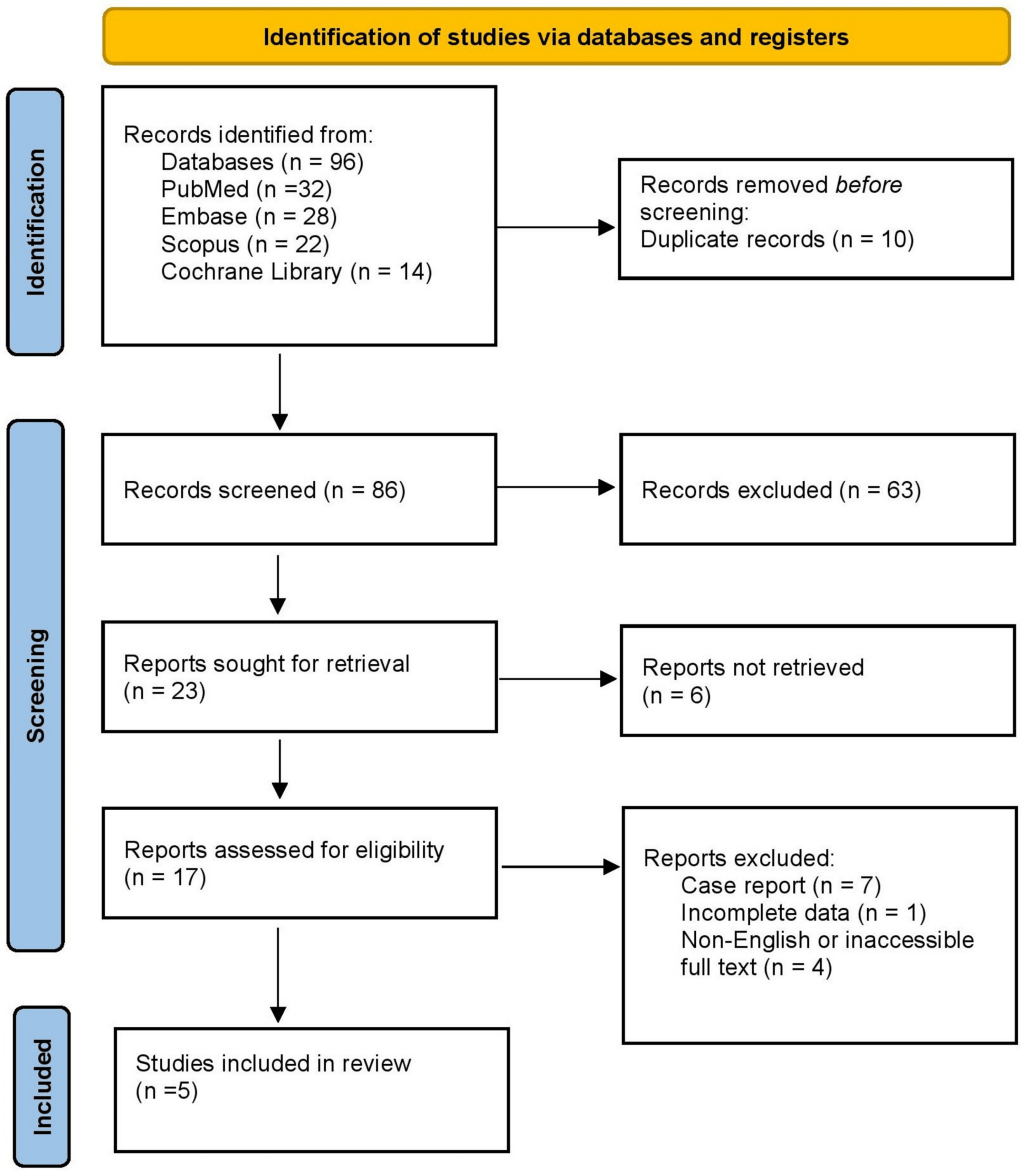
Preferred Reporting Items for Systematic Reviews and Meta-Analyses (PRISMA) 2020 flow diagram.

Figure 1 shows that the initial search identified a total of 96 records across the four databases. After removal of 10 duplicate entries, 86 unique records remained for title and abstract screening. Of these, 63 were excluded because they did not meet the inclusion criteria, leaving 17 articles for full-text assessment. Full-text review led to the exclusion of 12 studies - seven were case reports, one had incomplete data, and four were non-English or inaccessible full text. Ultimately, five studies fulfilled the eligibility criteria and were included in this systematic review. This process ensured that the final evidence base consisted of high-quality, relevant studies directly addressing the review question.

Characteristics of the Selected Studies

Table 1 summarizes the characteristics of five key studies evaluating head CT use in minor head injury. Stiell et al. assessed 3,121 Canadian ED patients with a GCS of 13-15 and demonstrated that the Canadian CT Head Rule had high sensitivity for clinically important TBI while reducing unnecessary scans [9]. Haydel et al. studied 520 US patients with a GCS of 15 and showed that the New Orleans Criteria effectively identified high-risk features requiring CT [10]. Shravat et al. audited 472 UK patients and found that, despite NICE guidelines, underuse and delayed CT scans persisted [11]. In a large cohort of 13,728 US patients, Mower et al. validated the NEXUS-II criteria, confirming its ability to safely exclude significant intracranial injury [12]. Sharp et al., analyzing 27,240 US patients, reported substantial CT overuse, highlighting gaps in the real-world application of the Canadian CT Head Rule [13]. Together, these studies demonstrate that while decision instruments are highly sensitive, variability in adherence contributes to both underuse and overuse of CT in clinical practice.

**Table 1:** Characteristics of the selected studies evaluating head CT use in minor head injury. MHI: minor head injury; mTBI: mild traumatic brain injury; ED: emergency department; GCS: Glasgow Coma Scale; CT: computed tomography; ciTBI: clinically important traumatic brain injury; CCHR: Canadian CT Head Rule; NOC: New Orleans Criteria; NICE: National Institute for Health and Care Excellence; NEXUS-II: National Emergency X-Radiography Utilization Study II

Studies	Population (P)	Exposure/condition (I)	Comparator (C)	Outcomes (O)	Pathophysiological findings	Anatomical impact	Clinical relevance
Stiell et al., 2001 [9]	3,121 adults with GCS of 13-15 after head injury in Canadian EDs	Application of the Canadian CT Head Rule (CCHR)	Physician judgment	CT yield, detection of clinically important TBI, neurosurgical intervention	Intracranial hemorrhage from blunt trauma	Skull fractures, intracranial bleeding	Demonstrated high sensitivity, reduced unnecessary CTs
Haydel et al., 2000 [10]	520 adults with GCS of 15 after blunt head trauma in US EDs	Application of New Orleans Criteria (NOC)	Clinical practice	Clinically important TBI on CT, hospital admission	Brain contusion, hemorrhage	Linear/non-displaced skull fractures	Identified high-risk clinical features for CT scanning
Shravat et al., 2006 [11]	472 ED patients across multiple UK centers with head injury	NICE head injury guidelines applied	Local practice	CT scan rates, adherence to guidelines	Post-traumatic bleeding risk	Intracranial hematoma detection	Showed underuse and delayed scans in some centers
Mower et al., 2005 [12]	13,728 US ED patients with blunt head trauma (GCS 15)	Application of NEXUS-II criteria	Physician discretion/existing practice	Identification of ciTBI, CT yield, and neurosurgical intervention rates	Intracranial hemorrhage, brain edema	Skull fractures, epidural, and subdural hematomas	Validated NEXUS-II as a highly sensitive rule for excluding ciTBI
Sharp et al., 2017 [13]	27,240 ED patients with minor head injury	Real-world CT ordering practices, evaluated against CCHR	Canadian CT Head Rule vs. actual CT use	Proportion of likely avoidable CTs, diagnostic yield, and guideline adherence	Post-traumatic intracranial bleed	Epidural, subdural, intraparenchymal hemorrhage	Showed substantial CT overuse; highlighted implementation gaps of CCHR

Risk of Bias Assessment

Table 2 presents the risk of bias assessment for the included studies using the Newcastle-Ottawa Scale (NOS). A study by Stiell et al. was rated low risk, supported by its large multicenter prospective design and rigorous validation of the Canadian CT Head Rule [9]. A study by Haydel et al. showed a moderate risk of bias due to its single-center setting and smaller sample size, though methods were robust [10]. A study by Shravat et al. demonstrated moderate-to-high risk given its retrospective audit design and variability in data capture [11]. Mower et al. reported a low-risk rating for NEXUS-II, supported by its validation in a large, multicenter US cohort and strong external validity [12]. A study by Sharp et al. was rated as having moderate risk of bias due to its retrospective design and reliance on administrative coding, despite a large sample size and strong real-world applicability [13]. Overall, the evidence base is strong, though study design limitations highlight areas for cautious interpretation.

**Table 2:** Risk of bias assessment of the included studies. CCHR: Canadian CT Head Rule; NOC: New Orleans Criteria; NICE: National Institute for Health and Care Excellence; NEXUS-II: National Emergency X-Radiography Utilization Study II; NOS: Newcastle-Ottawa Scale

Study	Study design	Risk of bias tool	Risk of bias rating	Justification
Stiell et al., 2001 [9]	Prospective multicenter cohort (derivation and validation of CCHR)	Newcastle-Ottawa Scale (NOS)	Low	Large sample, multicenter design, rigorous derivation and validation; minor selection bias possible from tertiary centers.
Haydel et al., 2000 [10]	Prospective single-center cohort (derivation of NOC)	NOS	Moderate	Smaller sample size, single-center limits generalizability, but robust methodology and outcome definitions.
Shravat et al., 2006 [11]	Multicenter audit of NICE guideline adherence	NOS (adapted for observational audit)	Moderate-high	Retrospective audit, variability in data capture across centers; useful real-world evidence but prone to reporting bias.
Mower et al., 2005 [12]	Prospective multicenter cohort (validation of NEXUS-II)	NOS	Low	Very large sample size, multicenter US EDs, independent validation; strong external validity.
Sharp et al., 2017 [13]	Retrospective observational cohort (US ED data compared with CCHR)	NOS	Moderate	Large dataset (27,240 patients), real-world applicability, but retrospective design and reliance on coding/records introduce bias.

Discussion

Computed tomography (CT) is the diagnostic standard for evaluating minor head injury (MHI) in the emergency department due to its ability to rapidly detect life-threatening intracranial pathology. Clinically important traumatic brain injury (ciTBI), though uncommon in MHI, carries significant morbidity and mortality if missed [14]. CT provides quick visualization of skull fractures, epidural or subdural hematomas, cerebral contusions, and diffuse brain swelling, thereby guiding neurosurgical decisions. However, widespread CT use raises important concerns, including high healthcare costs, resource utilization, prolonged ED crowding, and patient exposure to ionizing radiation, which is especially problematic in young patients because of cumulative cancer risks [15]. Thus, identifying which patients truly need imaging remains a critical issue in modern emergency medicine.

To refine decision-making, multiple evidence-based criteria have been developed. The Canadian CT Head Rule (CCHR) was designed for patients with GCS 13-15 and incorporates high-risk indicators such as failure to reach GCS 15 within two hours, suspected open or depressed skull fracture, basal skull signs, vomiting, or age ≥65 years, alongside medium-risk predictors like dangerous mechanism and amnesia ≥30 minutes [9]. The New Orleans Criteria (NOC), intended for fully alert patients with a GCS of 15, focuses on clinical symptoms including headache, vomiting, seizure, intoxication, persistent anterograde amnesia, and visible trauma above the clavicles [10]. The NEXUS-II decision tool identifies the absence of risk factors such as skull fracture, scalp hematoma, neurological deficit, abnormal alertness, coagulopathy, vomiting, and age ≥65 years, thereby providing a framework to safely exclude patients from CT imaging [12]. Meanwhile, NICE guidelines combine these approaches with explicit recommendations for time-to-scan targets, aiming to reduce both overuse and delays in indicated cases [11]. Collectively, these criteria demonstrate that structured clinical assessment can preserve sensitivity for ciTBI while improving specificity compared with unstructured physician judgment.

Evidence from large validation studies supports the performance of these rules. A study by Stiell et al. showed that CCHR achieved very high sensitivity for ciTBI and neurosurgical outcomes, outperforming physician judgment in identifying patients needing CT, and offered better specificity than NOC [9]. Haydel et al. found that while NOC also maintained 100% sensitivity, its lower specificity meant that more patients underwent CT unnecessarily, raising concerns about over-imaging [10]. Shravat et al., auditing UK hospitals, reported gaps in adherence to NICE guidelines, with delays and underuse of CT leading to potential risks of missed intracranial injury [11]. Mower et al., in a very large multicenter US study of over 13,000 patients, validated NEXUS-II and confirmed that it was safe and highly sensitive in identifying patients at risk of ciTBI [12]. Finally, a study by Sharp et al. demonstrated that despite the availability of validated rules, real-world CT ordering often exceeded recommendations, with a high proportion of scans deemed avoidable if CCHR were consistently applied [13]. Together, these findings highlight both the potential of decision rules to standardize care and the persistent challenges in translating evidence into practice.

Despite this strong body of evidence, limitations must be acknowledged. Many of the included studies were conducted in high-income countries with robust healthcare infrastructures, limiting generalizability to low-resource settings where CT availability is constrained. In addition, variations in clinical documentation, medico-legal concerns, and physician risk tolerance contribute to deviations from guideline-based practice. Elderly patients, those on anticoagulation, and individuals with comorbidities represent populations in which current rules may not perform optimally, often leading to more liberal imaging. Furthermore, retrospective designs, such as those discussed in a study by Sharp et al., carry an inherent risk of bias related to coding accuracy and incomplete data capture. These limitations underscore the need for ongoing prospective validation, targeted implementation strategies, and incorporation of clinical decision rules into electronic decision-support systems to improve adherence and ensure safe, efficient imaging practices in MHI.

## Conclusions

This systematic review demonstrates that head CT remains the cornerstone of evaluating minor head injury, but its use is often inconsistent with evidence-based criteria. Clinical decision rules, such as the Canadian CT Head Rule, New Orleans Criteria, NEXUS-II, and NICE guidelines, provide high sensitivity for detecting clinically important traumatic brain injury while reducing unnecessary imaging. Despite strong evidence, real-world practice shows both overuses, leading to avoidable radiation and cost, and underuse, risking delayed diagnosis. Variability stems from medico-legal concerns, physician judgment, and system-level barriers. Greater integration of validated rules into clinical workflows and decision-support systems can improve adherence and optimize patient safety. Future research should focus on refining these criteria for high-risk subgroups and ensuring implementation across diverse healthcare settings.

## References

[1] Maas AIR, Menon DK, Adelson PD (2017). Traumatic brain injury: integrated approaches to improve prevention, clinical care, and research. Lancet Neurol.

[2] Wintermark M, Sanelli PC, Anzai Y, Tsiouris AJ, Whitlow CT (2015). Imaging evidence and recommendations for traumatic brain injury: advanced neuro- and neurovascular imaging techniques. AJNR Am J Neuroradiol.

[3] Smith-Bindman R, Lipson J, Marcus R (2009). Radiation dose associated with common computed tomography examinations and the associated lifetime attributable risk of cancer. Arch Intern Med.

[4] Flanagan SR (2015). Invited commentary on "Centers for Disease Control and Prevention Report to Congress: traumatic brain injury in the United States: epidemiology and rehabilitation". Arch Phys Med Rehabil.

[5] Papa L, Stiell IG, Clement CM (2012). Performance of the Canadian CT Head Rule and the New Orleans Criteria for predicting any traumatic intracranial injury on computed tomography in a United States level I trauma center. Acad Emerg Med.

[6] Page MJ, McKenzie JE, Bossuyt PM (2021). Updating guidance for reporting systematic reviews: development of the PRISMA 2020 statement. J Clin Epidemiol.

[7] Eldawlatly A, Alshehri H, Alqahtani A, Ahmad A, Al-Dammas F, Marzouk A (2018). Appearance of population, intervention, comparison, and outcome as research question in the title of articles of three different anesthesia journals: a pilot study. Saudi J Anaesth.

[8] Wells Wells, George George, Beverley Beverley (2000). The Newcastle-Ottawa Scale (NOS) for assessing the quality of non-randomized studies in meta-analysis. https://www.scribd.com/presentation/461181619/oxford-web-ppt#:~:text=views39%20pages-,The%20Newcastle%2DOttawa%20Scale%20(NOS)%20For%20Assessing%20The%20Quality,designed%20for%20ease%20of%20use..

[9] Stiell IG, Wells GA, Vandemheen K (2001). The Canadian CT Head Rule for patients with minor head injury. Lancet.

[10] Haydel MJ, Preston CA, Mills TJ, Luber S, Blaudeau E, DeBlieux PM (2000). Indications for computed tomography in patients with minor head injury. N Engl J Med.

[11] Shravat BP, Huseyin TS, Hynes KA (2006). NICE guideline for the management of head injury: an audit demonstrating its impact on a district general hospital, with a cost analysis for England and Wales. Emerg Med J.

[12] Mower WR, Hoffman JR, Herbert M, Wolfson AB, Pollack CV Jr, Zucker MI (2005). Developing a decision instrument to guide computed tomographic imaging of blunt head injury patients. J Trauma.

[13] Sharp AL, Nagaraj G, Rippberger EJ (2017). Computed tomography use for adults with head injury: describing likely avoidable emergency department imaging based on the Canadian CT Head Rule. Acad Emerg Med.

[14] Uchiyama M, Mori K, Abe T, Imaki S (2024). Risk factors for clinically important traumatic brain injury in minor head injury in older people. Am J Emerg Med.

[15] Odle T (2020). Emergency computed tomography. Radiol Technol.

